# Cross-Sectional Detection of Acute HIV Infection: Timing of Transmission, Inflammation and Antiretroviral Therapy

**DOI:** 10.1371/journal.pone.0019617

**Published:** 2011-05-10

**Authors:** Cynthia Gay, Oliver Dibben, Jeffrey A. Anderson, Andrea Stacey, Ashley J. Mayo, Philip J. Norris, JoAnn D. Kuruc, Jesus F. Salazar-Gonzalez, Hui Li, Brandon F. Keele, Charles Hicks, David Margolis, Guido Ferrari, Barton Haynes, Ronald Swanstrom, George M. Shaw, Beatrice H. Hahn, Joseph J. Eron, Persephone Borrow, Myron S. Cohen

**Affiliations:** 1 Department of Medicine, University of North Carolina School of Medicine, Chapel Hill, North Carolina, United States of America; 2 Nuffield Department of Clinical Medicine, University of Oxford, Weatherall Institute for Molecular Medicine, Oxford, United Kingdom; 3 Blood Systems Research Institute, San Francisco, California, United States of America; 4 University of California San Francisco, San Francisco, California, United States of America; 5 Department of Medicine, University of Alabama at Birmingham, Birmingham, Alabama, United States of America; 6 Duke University, Durham, North Carolina, United States of America; University of Cape Town, South Africa

## Abstract

**Background:**

Acute HIV infection (AHI) is a critical phase of infection when irreparable damage to the immune system occurs and subjects are very infectious. We studied subjects with AHI prospectively to develop better treatment and public health interventions.

**Methods:**

Cross-sectional screening was employed to detect HIV RNA positive, antibody negative subjects. Date of HIV acquisition was estimated from clinical history and correlated with sequence diversity assessed by single genome amplification (SGA). Twenty-two cytokines/chemokines were measured from enrollment through week 24.

**Results:**

Thirty-seven AHI subjects were studied. In 7 participants with limited exposure windows, the median exposure to HIV occurred 14 days before symptom onset. Lack of viral sequence diversification confirmed the short duration of infection. Transmission dates estimated by SGA/sequencing using molecular clock models correlated with transmission dates estimated by symptom onset in individuals infected with single HIV variants (mean of 28 versus 33 days). Only 10 of 22 cytokines/chemokines were significantly elevated among AHI participants at enrollment compared to uninfected controls, and only 4 participants remained seronegative at enrollment.

**Discussion:**

The results emphasize the difficulty in recruiting subjects early in AHI. Viral sequence diversity proved accurate in estimating time of infection. Regardless of aggressive screening, peak viremia and inflammation occurred before enrollment and potential intervention. Given the personal and public health importance, improved AHI detection is urgently needed.

## Introduction

Acute human immunodeficiency virus (HIV) infection (AHI) constitutes the first stage of HIV infection. After HIV exposure local infection is established at transmission sites, and the virus spreads to regional lymph nodes and disseminates.[1] An expansion phase follows with exponential HIV replication in most individuals to peaks which can exceed 10 million copies/ml.[2] Subsequently, cell-mediated immune responses [3], [4], [5] lead to decreased viral replication.

The impact of events during AHI on the course of disease has been well-established[6], [7], [8] emphasizing the need for insight into virus-host interactions during AHI.[9] People with AHI represent a major risk for secondary HIV-1 transmission,[10], [11], [12] providing additional impetus for strategies to modulate AHI. However, AHI detection remains difficult, and only a small number of acutely infected subjects have been studied.

To find subjects immediately after HIV acquisition we employed cross-sectional screening to identify HIV RNA positive, seronegative subjects,[13] corresponding to the viral expansion phase.[14] At this time viral population *in vivo* has diverged minimally from the transmitted sequence(s), since few rounds of viral replication have occurred.[15] We correlated history of HIV exposure with HIV sequence diversity as a potential biomarker for date of infection. We examined plasma cytokine levels and the effects of antiretroviral therapy (ART) on these markers of inflammation. The results provide a clear, prospective picture of patients with AHI, and emphasize the difficulty in recruiting subjects very early in AHI due to missed opportunities for earlier diagnosis and diagnostic delays. Accordingly, wider awareness of the clinical presentation by medical providers as well as more rapid strategies to diagnosis AHI are needed.

## Methods

### Ethics Statement

This study and the additional studies in which participants could co-enroll on (see below) were approved by the University of North Carolina at Chapel Hill (UNC) and Duke University Institutional Review Boards. A separate informed consent was obtained for each study in which the subject participated.

### Recruitment

The state of North Carolina (NC) has an ongoing program to identify people in acute HIV infection (AHI) as part of its statewide HIV surveillance program. Since 2002, persons HIV-tested at approximately 135 publicly-funded sites have been included in the Screening and Tracing Active Transmission (STAT) Program.[13] Additional cases of acute HIV infection are identified through screening performed at primary care testing sites such as urgent care clinics, emergency departments, private doctor's offices and infectious disease clinics (Table 1), usually due to symptoms suggesting acute retroviral syndrome. Individuals meeting the following criteria from either referral source are referred: 1) EIA negative or indeterminate and positive NAT, 2) EIA indeterminate and positive EIA confirmation, and 3) EIA positive with seronegative documentation within the preceding 30 days. AHI diagnosis is the date of the first test detecting the presence of HIV, and not the date that the individual is notified of the result. When possible, screening samples were obtained for quantitative viral load and Western blot.

**Table 1:** Demographic and clinical characteristics of participants with acute HIV infection and seronegative controls.

Characteristic		AHI Participants	HIV Negative Controls	P value
Age, years; median (range)		32 (17–66)	34 (18–54)	0.5
Gender, N (%)	Male	33 (89.2)	17 (80.9)	0.4
	Female	4 (10.8)	-	
Risk category^a^, N (%)	Heterosexual	8 (21.6)	-	-
	MSM/Bisexual^b^	29 (78.4)	-	
Race, N (%)	Black	18 (48.7)	6 (28.6)	
	White	17 (46.0)	14 (66.7)	0.2
	Other	2 (5.4)	1 (4.8)	
STI at diagnosis, N (%)^c^	Yes^d^	7 (18.9)	4 (19.1)	0.99
	No	30 (81.1)	17 (80.9)	
Any symptom reported, N (%)	Yes	35 (94.6)	-	-
	No	2 (5.4)	-	-
Site of Diagnosis, N (%)				
	Health Department/STD Clinic	21 (56.8)	-	-
	ED/Urgent Care	7 (18.9)	-	-
	Primary Care Physician	7 (18.9)	-	-
	Other^f^	2 (5.4)	-	-
Enrollment CD4 T-cell count, cells/mm^3^; median (range)		541(13–1012)	-	-
Nadir CD4 T-cell count, cells/mm^3^; median (range)		487 (13–882)	-	-
HIV RNA at AHÍ diagnosis, copies/ml^e^ ; median (range)		592,690 (3,144-84,545,454)	-	-
Time from AHI diagnosis to enrollment, days; median (range)		16 (4–42)	-	-

a No participants endorsed injection drug use.

b MSM  =  Men who have sex with men.

c STI  =  sexually transmitted infection

d Syphilis (n = 2), gonorrhea (n = 1), Chlamydia trachomatis (n = 1), non-gonococcal urethritis (n = 1) and genital ulcer disease (n = 1). One female subject was diagnosed with concurrent bacterial vaginosus, pelvic inflammatory disease, trichomoniasis and genital ulcer disease.

e Viral Loads >750,000 copies/ml were included in the calculation of the medians as equal to 750,000 copies/ml unless sample was diluted and retested, in which case the true value was used.

f Other diagnosis sites included student health and drug treatment program

Following identification of suspected or confirmed acute HIV cases through rapid notification from the STAT program to NC DHHS surveillance or from primary care sites, specially trained NC DHHS Disease Intervention Specialists (DIS) perform the initial interviews, confirmatory tests and make referrals to care within 72 hours after receiving a report. In addition to standard information about the testing sites, reasons for HIV testing, demographics, HIV testing history and risk factors, DIS also collect detailed information about symptoms, risk behavior and partnerships for the STAT program [13]. Written informed consent for the use of personal de-identified information is obtained from all AHI participants.

### Enrollment

Suspected and confirmed AHI cases are referred for evaluation at UNC or Duke University, and can enroll in the Center for HIV/AIDS Vaccine Immunology (CHAVI) 001 Study: Acute HIV Infection Prospective Cohort if they meet AHI criteria defined as: 1) EIA negative and positive NAT; 2) positive EIA and positive NAT with a negative/indeterminate Western blot; 3) positive EIA, positive Western blot and EIA negative documentation within the preceding 45 days. Subjects can also co-enroll in a treatment study with co-formulated emtricitabine, tenofovir and efavirenz (Atripla) initiated within 30 days of AHI diagnosis and continued for 48 weeks.[16] Untreated acute subjects can enroll on another AHI longitudinal study. Sample collection begins at enrollment and at weeks 1, 2, 4, 8, 12, 16, 24, 36, 48, 60, 72, 84 and 96. Clinical and symptom data collected from all three studies were used in data analysis for this study. A separate informed consent was obtained for each study in which the subject participated. HIV negative control subjects were recruited via flyers placed at Duke Medical Center and the UNC Hospitals campus and local health departments. HIV negative controls did not differ from the AHI subjects in age, gender, race, or history of STIs using a non-parametric Mann-Whitney U-test (Table 1).

### Estimation of HIV risk exposure

To characterize estimated date of infection in AHI subjects, we reviewed charts to elicit exposure dates with all reported sex partners and partner HIV status in the 8 weeks preceding AHI diagnosis. Estimated-date-of-infection was defined as a function of the likelihood of the HIV source and timing of sexual contact with the suspected source. When available, single genome amplification (SGA) and DNA sequencing confirmed transmission partners. Acute retroviral syndrome (ARS) symptom data were reviewed to determine onset and resolution dates, including: fever, headache, night sweats, weight loss, myalgias, arthralgias, fatigue, rash, odynophagia, lymphadenopathy, oral candidiasis, mouth ulcers/sores, cough, loss of appetite, nausea/vomiting, diarrhea and abdominal pain in the 8 weeks before AHI diagnosis. With persistent lymphadenopathy or fatigue, ARS resolution date was the end date of all other symptoms. We present descriptive statistics of demographic characteristics and ARS-related symptoms.

### HIV sequence analysis

SGA and DNA sequence analysis were performed on the first available specimens for 24 of 37 participants. Approximately 10,000–20,000 viral RNA copies were extracted using the QIAamp viral RNA mini kit (Qiagen). HIV-1 RNA was reverse transcribed to cDNA using Superscript III Reverse Transcriptase (RT) System (Invitrogen.) The *env* gene was amplified using a limiting dilution approach,[17], [18], [19] to exclude *Taq* polymerase-induced errors and artifactual recombination, and to provide proportional representation of each viral sequence.[15], [20] PCR products were directly sequenced. To ensure sequences reflected single templates from *in vivo* populations, we excluded amplicons with “double peak” sequence chromatograms, indicating co-amplification of >1 template. DNA sequences from participants 1, 2, 3, and 4 were previously described in Keele *et al*.[15] Genbank accession numbers are: EU578952-EU578997, EU576425-EU576470, FJ496009-FJ496011, EU578998-EU579019, GU330247-GU331770, HQ908109-HQ908254.

DNA sequences were aligned using CLUSTAL W[21] and then codon-aligned manually.[21] Sequence differences were visualized using Neighbor-Joining trees (MEGA 4.0)[22] and *Highlighter* nucleotide transition and transversion plots (www.hiv.lanl.gov) to determine number of transmitted/founder viruses. G-to-A hypermutants from apolipoprotein B mRNA editing enzyme, catalytic polypeptide-like (APOBEC) signatures (www.hiv.lanl.gov) were excluded. Markov Chain Monte Carlo Simulation (MCMC) using Bayesian inference was used to estimate the infection date based on number of viral generations using BEAST (Bayesian Evolutionary Analysis by Sampling Trees v.1.5.3)[23] as previously described [24] with an HKY substitution model; HIV-1 generation time of 1.6 days; and a substitution rate of 2.16×10−5. Average tree model root height was used to determine the number of viral generations since infection with a 95% confidence interval (CI).

### Analysis of plasma cytokine and chemokine levels

Plasma levels of thirteen cytokines/chemokines (interleukin (IL)-1β, IL-2, IL-4, IL-5, IL-6, IL-7, IL-8, IL-10, IL-12(p70), IL-13, interferon (IFN)γ, granulocyte-macrophage colony-stimulating factor (GM-CSF) and tumor necrosis factor (TNFα) were measured at enrollment in the HIV-seronegative control subjects, and at time points from enrollment through week 24 in the AHI subjects using a high-sensitivity human cytokine Lincoplex kit (Millipore). Six factors (inducible protein [IP]-10, monocyte chemotactic protein [MCP]-1, monokine induced by IFNγ[MIG], macrophage inflammatory protein [MIP]-1α, MIP-1βand regulated on activation, normal T cell-expressed and -secreted [RANTES]) were measured by Bio-plex cytokine assay (Bio-Rad). Data were acquired on a Luminex-100 system and analyzed using Bio-Plex Manager software, v4.1 (Bio-Rad). Plasma levels of three cytokines were determined by ELISA: IL-15 using a high-sensitivity chemiluminescent assay (R&D Systems) and IFNβand IL-18 using colourimetric ELISAs (R&D Systems and Invitrogen, respectively). All samples were assayed in duplicate. Data on chemokine/cytokine elevations during AHI in plasma donors were used for comparison.[25]

Enrollment and week 16/24 chemokine/cytokine levels in AHI subjects were compared with those in the seronegative controls. Cytokine/chemokine levels in seronegative control subjects and treated and untreated AHI subjects were compared using a Kruskal-Wallis non-parametric test. These data were analyzed using Prism (version 4, GraphPad Software, Inc.) and R statistical software. Given the high chance of type 2 errors (false negative results) being incurred if p values were adjusted to correct for the number of comparisons made, the p values shown are raw rather than adjusted values. However, to reduce the occurrence of type 1 errors (i.e. concluding that chance observations were real) only test results with a p value of <0.01 were considered to be significant.

## Results

### Study Population

Between June 2006 and April 2008, 36 acutely infected participants were enrolled onto the CHAVI 001 study; 22 were referred from the NC STAT program and 14 were referred from community medical providers. Most were men who have sex with men (MSM) with a median age of 25 years (Table 1). The median viral load at diagnosis was 332,879 copies/ml and the median highest observed viral load was 592,690 copies/ml. For 81% of subjects, the initial viral load was the highest. The median CD4 count at enrollment was 541 cells/mm^3^ and median nadir CD4 count was 487 cells/mm^3^ occurring at enrollment in 20 (54%) subjects. Among 4 (11%) participants who remained seronegative at enrollment, the median maximum viral load was higher at 2,572,861 copies/ml, and the median CD4 count was lower at 327 cells/mm.^3^

Nearly all participants (95%) experienced ≥1 ARS-related symptom. The median number of symptoms was 5 (range 0–13.) Fever was most commonly reported (70%); approximately one-third had gastrointestinal symptoms and one-quarter reported odynophagia. The median symptom duration was 13 days (range 2–48). The median time from symptom onset to the highest observed viral load was 13 days (range -10–47 days). In linear regression analysis, number of symptoms (p = 0.03; 95% CI, 0.02–0.45) but not symptom duration (p = 0.7; CI, -0.07–0.05) was associated with higher log_10_HIV RNA.

Subjects had the option of beginning ART, and 27 (73%) elected to start treatment. Treated participants were older, median age of 32 (range 18–66) versus 22 years (range 17–56) among untreated acutes (p = .005). The number of ARS symptoms was higher among treated versus untreated acutes; median of 6 (range 1–13) versus 4 (range 0–8), respectively (p = .01). There were no statistically significant differences between treated and untreated participants regarding report of ≥2 symptoms, symptom duration, highest viral load or nadir CD4 count.

### Predicted time of acquisition of HIV

**Figure 1:**
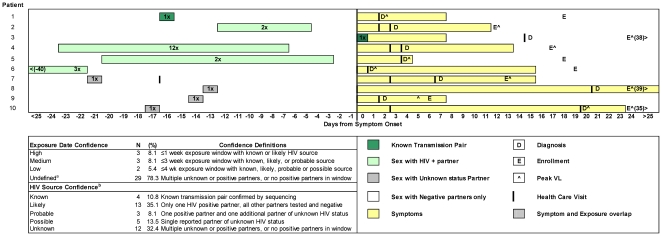
Timeline of AHI: estimated HIV exposure, symptom onset, presentation to care, AHI diagnosis and enrollment in subjects with a narrow window of exposure based on self-report. A narrow window of exposure is defined as all patients with high or medium exposure date confidence (*n* = 6), plus patients with a one-time exposure in the 8 weeks prior to diagnosis with a partner of unknown status (*n* = 4).

Given the desire to identify subjects as quickly as possible after infection, we attempted to estimate the date of exposure. Over one-third were evaluated at a medical facility prior to AHI diagnosis, representing missed opportunities for earlier diagnosis. Figure 1 depicts the timeline of exposures, symptom onset, presentation-to-care, AHI diagnosis and enrollment for subjects with a relatively narrow exposure window. From the date of blood draw, the mean turn-around time for NAT pooling results during the study period was 12 days (range 2 to 41). Four transmission pairs were confirmed by SGA and DNA sequencing; however, multiple sexual exposures occurred with two confirmed transmission partners (not shown), precluding establishment of an exposure date. Subject 3 (Figure 1) reported only one exposure on the date of enrollment. Subject 1 reported a one-time exposure with a confirmed transmission partner occurring 16 days before symptom onset. Using the midpoints of exposure windows for 7 of 10 subjects with relatively narrow exposure windows (subjects 3, 4 and 6 were excluded), median estimated exposure to HIV occurred 14 days before symptom onset (range 9–21 days, IQR 12–17).

The median time from symptom onset to initial presentation-to-care was 3.5 days (range -16–47); 3 participants developed symptoms after initial presentation. The median time from symptom onset to AHI diagnosis was 6 days (range -10–47) and to enrollment was 23 days (range 5–60). Notably, date of AHI diagnosis reflects the date of the first test result indicating AHI, and not when individuals are notified of their result. Accordingly, the delay from AHI diagnosis to enrollment reflects the time required to perform and confirm NAT pooling, trace and notify suspected AHI cases, and arrange for evaluation by a provider. There were no differences between median time from AHI diagnosis to enrollment between participants diagnosed through the NC STAT program and those referred from private providers. Among 4 seronegative participants at enrollment, median time from symptom onset to enrollment was shorter at 13 days (range 8–24). However, time from symptom onset to observed seroconversion among enrollment seronegatives was longer at 22 days (range 15–37) versus 17 days (range -10–41) among enrollment seropositives.

### Extent of viral sequence diversification

To confirm AHI status, viral diversification was assessed in 24 participants. On average, 29 full-length *env* amplicons were generated per subject. Given reasonable assumptions regarding viral mutation rate and generation time,[26] time since infection (time to most recent common ancestor [TMRCA]) was estimated using Bayesian molecular clock models. Recent publications highlight their use to model inter-continental HIV-1 spread,[27] subtype evolution,[28] and evolution during AHI.[26] We compared estimated-date-of-infection from SGA-derived sequences using BEAST to estimated-date-of-infection based on symptom onset, the latter defined as 14 days prior to the first ARS symptom (Table 2). Molecular clock models assume single variant transmission, as multiple variant transmissions overestimate TMRCA.[15], [26] Of 24 participants sequenced, 9 demonstrated multiple transmitted/founder viruses. Four of 9 subjects with multiple variants and 14 of 15 with single variants were self-reported MSM. As expected, BEAST overestimated TMRCA with multiple transmitted/founder viruses (not shown).

**Table 2:** Comparison of the estimated the date of infection based on Bayesian Evolutionary Analysis by Sampling Trees (BEAST) versus acute retroviral symptom onset.

Participant Number	Number of amplicons	BEAST days post-infection	Days post-infection (Symptoms)^d^
		(95% CI)^c^	
2	40	19 (7, 35)	15
3	45	38 (19, 63)	25
4	22	27 (11, 46)	17
6	19	31 (12, 54)	26
7	24	43 (15, 81)	51
10	44	21 (11, 33)	21
11	37	39 (19, 63)	33
14	11	23 (3, 50)	19
16	8	35 (6, 77)	26
19	17	7 (1, 18)	17
23	28	50 (22, 85)	29
24	23	21 (5, 42)	30
25	28	20 (7, 35)	18
29	36	69 (31, 116)	41
31	38	49 (28, 73)	32

a Symptom onset minus 14 days.

b Sample date minus BEAST estimated days post-infection.

c BEAST estimated days post-infection from time of sampling.

d Sample date minus symptom estimated infection date.

**Figure 2:**
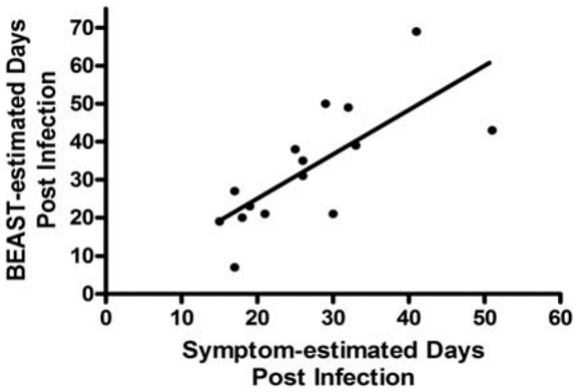
Estimated dates of infection based on symptom onset (x axis) vs. BEAST (y axis) in 15 patients with single variant transmissions. Infection dates were inferred by subtracting 14 days from symptom onset and compared to BEAST calculations relative to sampling of blood plasma for SGA *env* sequence generation. The slope of the line is 1.166±0.2995 with R^2^ = 0.54 for all 15 patients.

BEAST estimates of single variant transmissions (±95% CI) overlapped with estimated-date-of-infection based on symptoms. The interval between infection and participant sampling per symptom onset ranged from 17–51 days (mean 27). Similarly, estimated TMRCA using BEAST ranged from 7–69 days (mean 33). Estimated-dates-of-infection based on symptoms versus BEAST had a positive correlation with R^2^ = 0.54 for all 15 participants (Figure 2). Analyzing 14 participants with estimated-dates-of-infection within 4 weeks of symptom onset increased the correlation coefficient (R^2^ = 0.72), suggesting BEAST estimates are most accurate when combined with more recent clinical data, possibly due to recall bias.

### Plasma cytokine and chemokine levels

**Figure 3:**
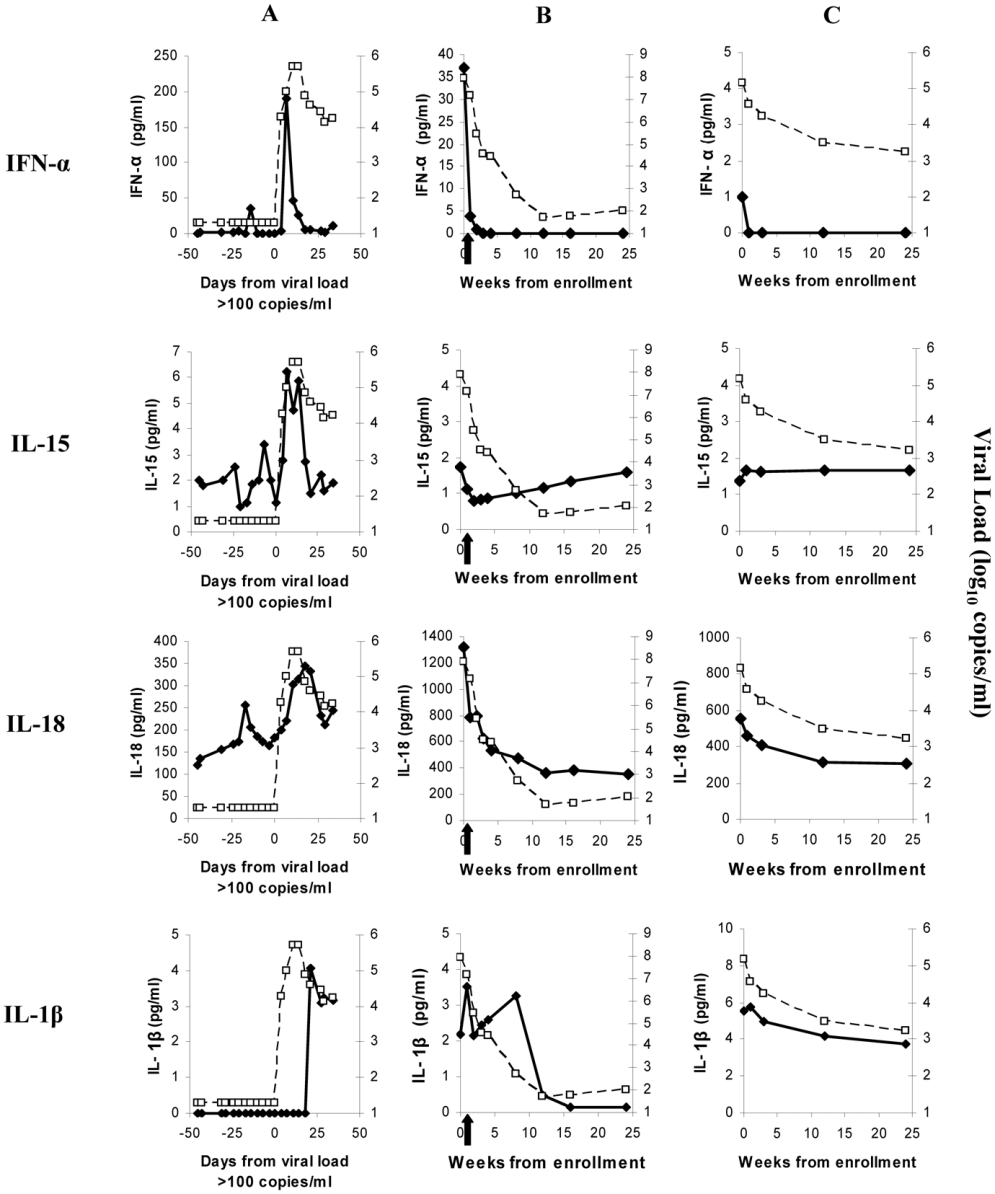
Viral loads and plasma levels of IFNα, IL-15, IL-18 and IL-1βin sample time courses from three subjects acutely infected with HIV. A is a US plasma donor, whose sample time course is plotted in days and is aligned relative to the time (designated day 0) when the plasma viral load first reached 100 copies/ml (i.e. the start of the viral expansion phase). B and C are CHAVI 001 subjects, whose sample time courses are plotted in weeks, and are aligned relative to the time of study enrollment (week 0). Subject B started ART just after study enrollment, as indicated by the black arrow. Viral load data is plotted as open squares joined by dotted lines, and is expressed as log_10_ RNA copies/ml. Data for each cytokine is plotted as filled symbols joined by solid lines, and is expressed as pg/ml.

Prior analysis of plasma donor samples from eclipse and viral expansion phases demonstrated cytokine/chemokine elevations as viremia increased.[25] Similar analysis of plasma cytokine/chemokine levels was performed on sequential samples from 23 CHAVI AHI subjects. Figure 3 shows examples of data for four cytokines in one plasma donor [25] and two CHAVI AHI subjects. Elevations in cytokines such as IFN-α and IL-15, transiently up-regulated before peak viremia,[25] were rarely captured in CHAVI AHI subjects where enrollment sometimes occurred prior to/near peak viremia (subject B), but usually occurred during the viral contraction phase (subject C). Elevations were more commonly observed in analytes with rapid but sustained increases during AHI, such as IL-18; or in analytes up-regulated at/after peak viremia, such as IL-1β. This was emphasised by comparing enrollment analyte levels in 22 AHI subjects (one enrollment sample was missing) and 21 CHAVI HIV-seronegative controls (Table 3). Only 10 analytes were significantly higher (p<0.01) in AHI participants: IP-10 and IL-18, which exhibit rapid, sustained increases in AHI;[25] IL-6, IL-10 and IFNγ, which also exhibited sustained, although somewhat slower, increases in AHI in the plasma donors; MIG, not analyzed in the plasma donors; and IL-1β, IL-2, IL-7 and GM-CSF, which showed relatively late increases in approximately half of plasma donors (where sampling frequently ended around peak viremia.)[25] Although most AHI participants were not enrolled early enough to capture cytokine elevations during viral expansion, this cohort provides a complementary picture of analyte levels later in AHI.

**Table 3:** Median plasma levels of 22 cytokines and chemokines at enrollment and week 16–24 in CHAVI 001 AHI subjects and HIV-seronegative controls.

Analyte	HIV negative enrollment median (IQR)^a^	AHI enrollment	P - enrollment^c^ (95% CI)	AHI week 16–24	P - week 16–24^c^
		median (IQR)^b^		median (IQR)^d^	(95% CI)
IL-1β	0.20 (0.07–0.93)	3.57 (0.50–7.73)	0.0003 (0.39–5.45)	2.07 (0.11–7.73)	0.015 (<0.01–5.39)
IL-2	3.72 (1.03–6.07)	19.94 (11.41–63.92)	0.0001 (8.57–49.32)	29.7 (1.00–56.56)	0.040 (<0.01–39.70)
IL-4	64.29 (6.36–241.04)	106.58 (18.40–244.98)	0.496 (-60.21–96.17)	72.07 (8.08–175.62)	0.778 (-107.04–65.24)
IL-5	0.67 (0.07–1.22)	1.13 (0.32–2.40)	0.324 (-0.27–1.10)	0.6 (0.26–2.43)	0.660 (-0.50–0.92)
IL-6	4.02 (0.66–16.91)	24.91 (11.89–34.03)	0.003 (6.18–25.98)	13.63 (2.88–33.32)	0.139 (-0.59–22.93)
IL-7	1.10 (0.32–2.93)	7.56 (0.98–14.31)	0.004 (0.65–10.21)	7.0 (0.76–18.19)	0.031 (<0.01–11.57)
IL-8	4.66 (2.02–10.32)	8.28 (3.54–13.34)	0.185 (-1.32–6.95)	4.19 (0.77–15.01)	0.933 (-3.40–7.10)
IL-10	5.26 (2.82–10.83)	19.61 (11.10–45.92)	0.002 (5.08–25.56)	18.63 (3.39–31.75)	0.317 (-2.50–21.35)
IL-12 (p70)	1.82 (0.92–6.25)	11.9 (1.68–34.93)	0.080 (-0.03–23.70)	11.45 (1.58–32.43)	0.167 (-0.52–21.33)
IL-13	36.49 (6.83–125.47)	49.40 (23.63–101.36)	0.827 (-38.99–38.76)	28.84 (4.69–66.58)	0.414 (-61.83–18.13)
IFNγ	2.24 (1.60–5.43)	14.55 (4.94–39.59)	0.004 (1.31–24.88)	14.72 (1.60–51.08)	0.043 (<0.01–28.90)
TNFα	4.67 (3.48–6.38)	7.33 (5.22–9.77)	0.060 (-0.12–4.12)	7.42 (3.81–8.85)	0.172 (-1.26–3.83)
GM-CSF	4.72 (2.35–12.60)	15.43 (5.50–50.54)	0.009 (1.42–33.63)	4.32 (1.25–18.21)	0.810 (-2.45–11.39)
MCP-1	41.04 (27.36–57.98)	43.77 (29.64–82.15)	0.671 (-16.40–24.72)	35.67 (10.24–72.66)	0.490 (-27.33–15.67)
MIG	295.85 (173.71–453.99)	1164.01 (391.36–1926.59)	<0.0001 (286.18–1522.60)	353.35 (115.31–1031.00)	0.564 (-126.17–536.19)
MIP1α	n/c^e^	n/c	n/c	n/c	n/c
MIP1β	36.81 (30.69–65.81)	33.88 (20.18–49.09)	0.123 (-25.71–2.98)	23.48 (14.28–46.86)	0.041 (−27.96–(−0.84))
RANTES	300.28 (223.41–985.06)	416.22 (261.53–749.84)	0.971 (-238.57–195.57)	298.78 (152.37–396.11)	0.172 (-583.07–57.67)
IP-10	253.05 (177.16–334.77)	470.5 (286.56–718.71)	0.003 (72.13–359.48)	150.32 (97.61–357.42)	0.172 (-149.20–33.62)
IFNα	1.563^f^ (1.56–1.56)	6.64 (1.56–96.75)	0.024 (<0.01–42.4)	1.56 (1.56–46.5)	0.122 (<0.01–16.4)
IL-18	161.60^f^ (90.32–270.99)	555.5 (316.07–883.01)	0.0001 (198.55–635.24)	390.17 (262.25–511.40)	0.001 (104.76–357.47)
IL-15	0.84^g^ (0.79–0.89)	1.26 (0.92–1.61)	0.035 (<0.01–0.58)	1.15 (0.89–1.51)	0.04 (0.02–0.6)

a Median analyte levels (pg/ml) in 21 HIV-seronegative control subjects. The 25^th^ and 75^th^ percentiles of the range are also given (IQR).

b Median analyte levels (pg/ml) in 22 AHI subjects. The 25^th^ and 75^th^ percentiles of the range are also given (IQR).

c Analyte levels in the seronegative controls at enrollment were compared to analyte levels in the AHI subjects at enrollment and week 16–24 using a Mann-Whitney U test. The p values are shown; significant values (p<0.01) are highlighted in bold. 95% confidence intervals are also shown (95% CI).

d Median analyte levels (pg/ml) in 18 AHI subjects. The 25^th^ and 75^th^ percentiles of the range are also given (IQR).

e n/c  =  not calculated (MIP-1α levels in the majority of samples were below the limit of assay detection).

f For IFNα and IL-1,8 n = 14.

g For IL-15, n = 10.

This cohort also provided an opportunity to determine levels at which plasma analytes stabilise in AHI. Comparison of analyte levels at week 16/24 post-enrollment in 18 acute subjects (11 of whom started ART shortly after enrollment and 7 of whom remained untreated) with those in the 21 seronegative controls sampled at enrollment (Table 3) showed that IL-18 was significantly elevated (p<0.01) among AHI subjects, whilst levels of several other analytes (IL-1β, IL-2, IL-7, IL-15 and IFNγ) exhibited differences of lower significance (p<0.05) between the two groups, and others (IL-6, IL-10 and IL-12) trended toward higher levels in AHI subjects, possibly failing to reach statistical significance because of small group sizes. In contrast to other analytes that were elevated in acute subjects versus controls at 16–24 weeks, levels of MIP-1βwere slightly reduced (p<0.05). These results indicate that systemic levels of many cytokines/chemokines stabilise in the near normal range after the AHI cytokine storm, but a subset remain elevated into early infection.

**Figure 4:**
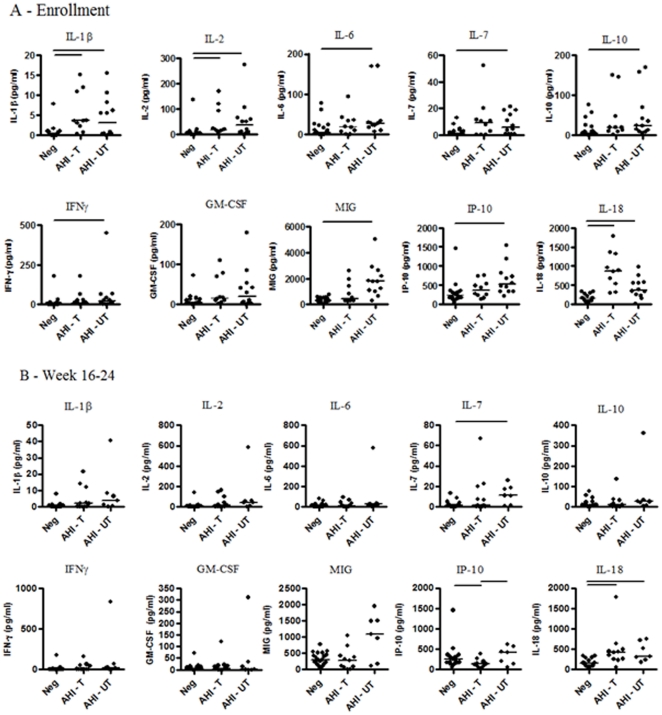
Comparative analysis of plasma levels of 10 selected analytes at enrollment and week 16–24 in AHI subjects commencing ART, AHI subjects choosing not to start ART and HIV-seronegative controls. Plasma levels of 22 cytokines and chemokines were measured in sample time courses from a total of 23 AHI subjects and 21 HIV seronegative controls (Neg). Data for ten analytes (IL-1β, IL-2, IL-7, IFNγ, GM-CSF, MIG, MIP-1β, IP-10, IL-18 and IL-15, each expressed as pg/ml plasma) is shown at A) the enrollment time point (prior to commencement of therapy), when samples were available from 10 CHAVI 001 AHI subjects who chose to commence ART after the enrollment time point (AHI - T) and 12 AHI subjects who chose to remain untreated (AHI - UT) and B) week 16 or 24, when samples were available from 11 CHAVI 001 AHI subjects who chose to commence ART after the enrollment time point (AHI - T) and 7 AHI subjects who chose to remain untreated (AHI - UT). In both A and B, data are also shown from 21 HIV-seronegative subjects (sampled at the enrollment time point), except for IL-18 where n = 14 and IL-15 where n = 10. Each symbol represents data for an individual subject, and horizontal lines represent the median analyte level in the subject groups. Bars at the top of each graph show statistically significant differences between groups (p<0.01; Kruskal-Wallis non-parametric test).

To evaluate whether severity of AHI symptoms influenced ART initiation, possibly related to the magnitude and/or duration of cytokine/chemokine levels, we compared enrollment analyte levels in 21 seronegative participants, 10 treated AHI participants, and 12 untreated AHI participants. Data for the 10 analytes significantly elevated at enrollment in AHI participants compared to seronegative controls are shown (Figure 4A). There was a trend towards higher IL-18 levels and lower MIG and IP-10 levels in treated versus untreated participants, although no difference between these groups reached statistical significance, possibly due to the low sample sizes.

We also compared analyte levels between seronegative controls (at enrollment) and treated and untreated AHI subjects at week 16–24. Data for 10 analytes is shown (Figure 4B). Only IP-10 differed significantly (p<0.01) between treated and untreated participants at week 16/24, although there were also trends for levels of other analytes, including IL-7 and MIG, to be higher in untreated AHI participants than in treated subjects that did not reach statistical significance, again likely due to the small number of subjects per group.

## Discussion

AHI is a critical phase of infection during which irrevocable damage to the immune system[29], [30] and substantial secondary transmission occurs.[10], [11], [12] The goal of this study was to identify AHI participants using cross-sectional screening, and to characterize clinical, immunological and virological evolution of AHI detected via this strategy. The results demonstrate the challenge in finding and rapidly enrolling patients very early in AHI, as only 4 participants remained seronegative at enrollment. The time from symptom onset to seroconversion was longer among those remaining seronegative at enrollment, suggesting seropositivity may be an inaccurate marker of the degree of “acuteness.” We anticipated cross-sectional screening would identify early HIV acquisition and many participants would be asymptomatic. However, nearly all participants were symptomatic and sought care and/or HIV testing due to symptoms. Indeed, over one-third were seen at least once prior to diagnosis, reflecting symptom non-specificity and that the clinical suspicion and risk assessment for AHI remains low among practitioners. Notably, the overall delay from symptom onset to the date of testing for AHI was relatively short among participants (median 6 days) compared to the delay from date of testing for AHI to enrollment (median 16 days). The majority of the latter delay results from a median turn-around of 12 days for confirmed NAT pooling results.

In this study, we used sequencing to examine viral diversification and confirmed the short duration of infection. Using SGA to estimate infection dates based on number of viral generations, BEAST results correlated well with estimated-date-of-infection per symptom report in subjects with single variant transmissions. To our knowledge, this is the first report of using molecular clock modeling in conjunction with symptom data to estimate date of HIV acquisition. The results validate using molecular clocks to confirm AHI with clinical data, and to estimate HIV transmission dates without specific exposure or symptom information. Findings are consistent with prior studies with well-described HIV exposure dates,[31], [32] and support use of 14 days prior to symptom onset to estimate HIV transmission dates.

Previous retrospective study of samples obtained from frequent plasma donors before, during and after HIV acquisition demonstrated elevations in multiple cytokines/chemokines during viral expansion.[25] A similar cytokine storm was not observed in this prospective study, suggesting enrollment occurred after peak levels of viremia and cytokines. However, differences were observed between the AHI subjects and seronegative controls in levels of some cytokines/chemokines that are elevated for a relatively sustained period during the acute phase (i.e. IL-18, IP-10, IFNγ, IL6 and IL-10) and in other analytes that are increased later in AHI (i.e. IL-1β, IL-2, IL-7 and GM-CSF). In line with these observations, a recent study in a high risk female cohort reported significant elevations in IL-1, IL-2, IL-7 and IP-10 at a median of 6 weeks post-HIV-infection.[33] More pronounced differences are likely not demonstrated in our cohort due to the delay in confirming AHI diagnosis as above.

Increased immune activation and cytokine levels during AHI are likely instrumental in inducing clinical symptoms, which typically begin during viral expansion when there are very high circulating levels of cytokines and chemokines.[25] In our study, symptoms frequently persisted after the peak in viremia and cytokine levels; however, some analytes including IL-1β(an endogenous pyrogen) were elevated at enrollment (coinciding with the viral contraction phase) and may have contributed to the prolonged symptom duration observed in this cohort. It seemed possible that symptom severity would influence subjects' decision to commence ART. Although the number of symptoms reported by participants who initiated ART was higher, there were no statistically significant differences in those with and without ART. However, the small number of participants in the groups limits this analysis, and other factors (age) may also play a significant role. We observed no significant difference in plasma cytokine/chemokine levels at enrollment in treated versus untreated subjects, but cannot exclude that more intense cytokine responses may have occurred before enrollment in treated subjects.

ART in early HIV has not demonstrated substantial long-term clinical benefit;[34], [35] however, studies suggest early ART could reduce the viral reservoir[36], [37] and HIV transmission.[38] We noted minimal effect of ART on levels of cytokines/chemokines at week 16–24 possibly due to the delay from presentation-to-care, to diagnosis and for referral. Accordingly, ART typically commenced after peak viremia, when the cytokine storm and viral replication were subsiding. However, analysis of the effects of ART initiation in AHI on levels at which cytokines/chemokines stabilize in early infection was not the primary goal of this study, and it remains possible that we would have detected more differences in cytokine/chemokine levels in ART-treated and untreated subjects if our subject groups had been larger. Previous studies have reported that ART initiation in chronic infection is associated with a decline in circulating levels of cytokines/chemokines including IL-1β, IL-6, IL-10, TNFα and alpha/beta chemokines [39], [40], although other studies reported conflicting findings, such as no effect of ART on circulating levels of IL-6 and C-reactive protein.[41], [42] Further work is required to address whether these divergent results may be attributable to effects of the timing of ART initiation (during acute, early chronic or later-stage infection).

It is regrettable that AHI detection in the general population remains uncommon since interventions during AHI could lead to both personal and public health benefit, with as many as 39% of new HIV cases ascribed to untreated and unrecognized AHI.[43] Our findings utilizing cross-sectional screening to detect AHI emphasize that more expedient diagnostic methods for detection of AHI are urgently needed, such as point-of-care virological assays and/or algorithms incorporating 4^th^ generation EIA assays. More rapid diagnosis of AHI with rapid entry into care following detection would allow individuals to fully benefit from treatment and secondary prevention, and the study of the earliest immunological events in AHI to inform treatment and vaccine strategies. In the interim, formal guidelines for the management of such patients await further results of clinical and translational studies.[44]
